# Treatment Efficacy of Imidocarb Dipropionate in Saanen Goats Experimentally Infected with *Babesia aktasi*

**DOI:** 10.3390/pathogens15080833

**Published:** 2026-08-10

**Authors:** Mehmet Can Ulucesme, Sezayi Ozubek, Munir Aktas

**Affiliations:** Department of Parasitology, Faculty of Veterinary Medicine, University of Firat, Elazığ 23200, Türkiye; sozubek@firat.edu.tr (S.O.); maktas@firat.edu.tr (M.A.)

**Keywords:** caprine babesiosis, *Babesia aktasi*, hematological parameters, parasitemia, chemotherapy

## Abstract

*Babesia aktasi* is a recently discovered species that is highly prevalent in native goats in Türkiye’s Mediterranean region. Although it does not induce clinical disease in local breeds, it causes severe illness in Saanen goats, manifesting with high fever, anemia, hemoglobinuria, and jaundice. Imidocarb dipropionate (IMDP) has been reported to be therapeutically effective against *Babesia* species. This study aimed to evaluate the therapeutic efficacy of IMDP and its ability to eliminate the parasite in experimentally infected Saanen goats. Twelve goats were assigned to treatment and control groups (*n* = 5 per group) and infected using fresh blood from two splenectomized donors. All goats developed clinical babesiosis, with parasitemia ranging from 3.8% to 22. Semi-nested PCR specific to *B. aktasi* was performed for 30 days post-treatment. Following treatment with IMDP (1.2 mg/kg), clinical signs resolved by the third and fourth days, and parasitemia became microscopically undetectable. However, one goat in the treatment group and four goats in the control group died shortly after infection. Hematological parameters (HCT, RBC, HB) decreased during infection but normalized in surviving animals. Notably, *B. aktasi* DNA remained detectable by PCR up to 30 days after treatment. These findings indicated that IMDP resolved clinical signs and eliminated microscopically detectable parasitemia; however, it may not completely eliminate the parasite at the molecular level in *B. aktasi* infections. The results of this study provide valuable information for optimizing therapeutic approaches and improving our understanding of treatment responses in new species *B. aktasi*.

## 1. Introduction

Babesiosis, a zoonotic disease, is an infection caused by hemoprotozoan parasites of the genus *Babesia*. The disease causes severe infections in various domestic and wild animals, particularly in ruminants from tropical-subtropical regions, and it results in significant economic losses [1,2,3,4,5]. Various *Babesia* species have been identified as responsible for causing ovine babesiosis [6]. Of these, *B. ovis* is considered the main and most virulent species, especially in sheep [7,8,9]. In recent years, progress in molecular parasitology has facilitated the discovery of several new species and genotypes. These include *Babesia* sp. Xinjiang, *B. motasi*-like complex, and *Babesia lengau*-like in sheep [10,11,12,13], as well as *Babesia aktasi*, which was recently described in goats [14].

*Babesia aktasi* was first identified using molecular methods by Ozubek et al. [15] in indigenous goats. A subsequent survey revealed that this parasite is widespread in clinically healthy goats [16]. Pathogenicity studies have shown that although *B. aktasi* causes subclinical infections in immunocompetent indigenous goats, it can lead to severe clinical disease in immunosuppressed goats [17]. To better understand host susceptibility, pathogenicity trials were conducted in purebred Saanen goats to evaluate the parasite’s behavior in a different breed of the same host species. These studies revealed that *B. aktasi* is highly pathogenic in Saanen goats, causing severe clinical babesiosis resulting in death [18]. In contrast, experimental infections in sheep have shown no evidence of susceptibility to *B. aktasi*, suggesting a species-specific resistance [19].

Imidocarb dipropionate (IMDP) is one of the most commonly preferred antiprotozoal drugs for the treatment of babesiosis [20,21]. Introduced in the 1970s for the treatment of bovine babesiosis, IMDP, an aromatic diamidine derivative, has been reported as the only babesicidal agent capable of completely eliminating the parasite from the host [22,23]. Although the exact mechanism of action is still debated, IMDP is thought to block the uptake of inositol into *Babesia*-infected erythrocytes, leading to starvation of the parasite [24]. IMDP has demonstrated high therapeutic efficacy against *B. ovis*, which is pathogenic in sheep, by reducing both parasitemia (PPE) and clinical signs, and its prophylactic effect has also been demonstrated [23,25,26]. However, it has shown limited efficacy against other species such as *Theileria equi* and the newly identified *T. haneyi*, with persistent infections observed despite treatment [27]. Similarly, in the treatment of *Babesia negevi*, a newly described species in dogs, IMDP has been reported to reduce clinical symptoms but has failed to completely eliminate the parasite [28].

Differences in the efficacy of antibabesial drugs among *Babesia* species have been reported in both bovine and canine babesiosis [29,30]. The first specific drug used against bovine babesiosis, trypan blue, was highly effective against *B. bigemina* infections but was reported to be ineffective against *B. bovis* [29]. Similarly, *Babesia* species infecting dogs differ in their susceptibility and response to antibabesial drugs. The *Babesia canis* and *B. rossi* are generally susceptible to IMDP and diminazene aceturate, whereas *B. gibsoni* and *B. conradae* are relatively resistant to these drugs, and combination therapy with atovaquone and azithromycin is generally recommended [30]. These situations demonstrate that the therapeutic efficacy observed against one *Babesia* species, or even a single strain, cannot be directly extrapolated to another species.

In this study, the therapeutic efficacy of IMDP was investigated in experimental infections with *B. aktasi*, which is known to be highly pathogenic in Saanen goats. Determining treatment efficacy is essential for establishing an evidence-based treatment strategy against *B. aktasi* and for contributing to a better understanding of its biology, thereby providing valuable information for the therapeutic management of this recently described and highly pathogenic species.

## 2. Materials and Methods

### 2.1. Ethical Statement

All experimental procedures involving animals were conducted in accordance with the applicable Turkish legislation on the protection and welfare of animals. The study protocol was reviewed and approved by the Animal Experiments Local Ethics Committee of Fırat University (Approval No. 2021/04).

### 2.2. Type of Parasite

*Babesia aktasi*, preserved in the cryobank stocks of the Department of Parasitology, was used for experimental infection in this study.

### 2.3. Selection of Experimental Animals

**Figure 1:**
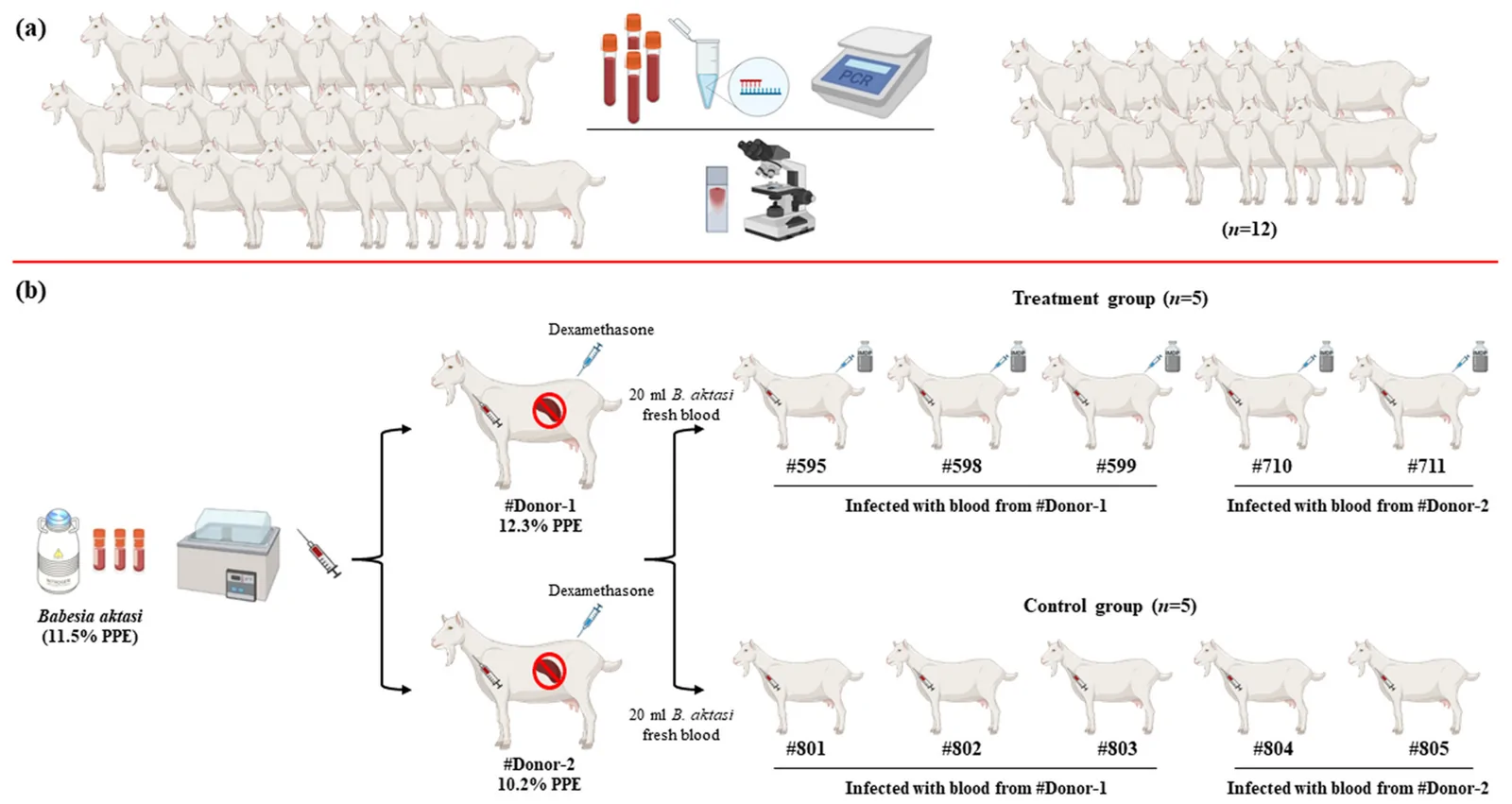
Graphical representation of the experimental process. (**a**) Selection and determination of *Babesia*, *Theileria*, and *Anaplasma* spp.-free Saanen goats; (**b**) infection of the donor and Saanen goats in experimental groups (treatment and control groups) with *B. aktasi* cryostabilate (PPE 11.5%) and fresh infected blood (PPE 12.3% and 10.2%), respectively.

In this study, purebred Saanen goats (*n* = 12) confirmed to be free of *Theileria*, *Babesia*, and *Anaplasma* species were included. To select the experimental animals, blood samples and peripheral blood smears were collected from 5- to 6-month-old purebred male Saanen goats belonging to a breeder in a rural area in the vicinity of Elazig province. A volume of 2 mL of blood samples was collected from each animal using vacutainer tubes containing EDTA-K_2_. The collected samples were transported to the laboratory under appropriate storage conditions. The isolated DNA samples (PureLink™ Genomic DNA Mini Kit, Invitrogen, Carlsbad, CA, USA) were analyzed for the presence of *Theileria*, *Babesia*, and *Anaplasma* species using nested PCR [16,18,31]. All twelve purebred Saanen goats (#Donor-1, #Donor-2, #595, #598, #599, #710, #711, #801, #802, #803, #804, #805) were determined to be negative for these species by nested PCR (nPCR) (Figure 1a). The goats were purchased and transferred to the small ruminant paddocks of the Elazığ Veterinary Control Institute. Each goat was maintained individually in a tick-free unit and allowed ad libitum intake of feed and water.

### 2.4. Experimental Design and Splenectomy

The purebred Saanen goats were divided into 2 groups: a control group consisting of 5 animals (#801, #802, #803, #804, #805) and a treatment group also comprising 5 animals (#595, #598, #599, #710, #711). Two Saanen goats were used as donors (#Donor-1 and #Donor-2) for the experimental infection of the goats in both groups (Figure 1b). The number of animals used in each group (*n* = 5) was determined based on ethical guidelines complying with the 3Rs principle (Replacement, Reduction, and Refinement) in animal experimentation. To obtain scientifically valid results without compromising the integrity of the experiment, the minimum number of animals was used to reduce the number of animals used. This approach is aligned with previous experimental studies involving *Babesia* spp., in which five animals from each group were generally adopted as a standard [32,33,34].

The two selected donors (#Donor-1 and #Donor-2) were transported to the Animal Hospital in Fırat University, where a splenectomy was performed by experienced veterinary surgeons using standard anesthesia, analgesia, and surgical procedures [17,26]. Following the splenectomy, the donors relocated to a tick-free unit, where they underwent routine post-operative care [35]. Three weeks after the splenectomy, the donors were re-tested by microscopic examination and nPCR, confirming that they were free from *Theileria*, *Babesia*, and *Anaplasma* species.

### 2.5. Experimental Infection of Donor Goat with B. aktasi Stabilate

For experimental infection, the cryostabilate with 11.5% Percent Parasitized Erythrocytes (PPE) was thawed at 37 °C, and 15 mL was intravenously inoculated into the donors (Figure 1b). Following the stabilate inoculation, the donors were administered 20 mg/day of dexamethasone for three days (Vetakort^®^ 4 mg, Vetas, Istanbul, Türkiye) as previously described [11,17]. Clinical and parasitological findings of the donor goats were monitored daily. On day 7 post-infection (DPI), parasitemia in #Donor-1 reached 12.3%. In #Donor-2, parasitemia reached 10.2% on day 8 post-infection. A total of 200 mL of blood was collected from the two donor goats at these time points. Subsequently, both donor goats were treated with a single dose of IMDP (1.2 mg/kg, Imicarp, Teknovet, Istanbul, Türkiye), followed by oxytetracycline (10 mg/kg, Primamycin/LA^®^, Zoetis, Parsippany, NJ, USA) administered three times on alternate days.

### 2.6. Experimental Infection of Saanen Goats

The Saanen goats in the treatment (#595, #598, #599, #710, #711) and control (#801, #802, #803, #804, #805) groups were experimentally infected by intravenous inoculation of 20 mL of infected blood. Following inoculation, the goats were monitored throughout the course of clinical infection. Blood samples were collected from all goats throughout the experimental period for microscopic examination, DNA isolation, and hematological analyses.

### 2.7. Treatment

A single intramuscular dose of 1.2 mg/kg of IMDP (Imicarp, Teknovet, Istanbul, Türkiye) was administered to the treatment group. The timing of treatment was based on clinical observations rather than hematological or microscopic findings, in order to reflect field conditions and to mirror the decision-making process typically used in practice. Accordingly, treatment was initiated when clinical signs such as anorexia, lethargy, weakness, or recumbency were observed.

### 2.8. Microscopic Examination, Genomic DNA Isolation, and Assessment of B. aktasi Presence Post-Treatment with IMDP

Peripheral blood slides prepared from the Saanen goats were fixed with absolute methanol and stained with 10% Giemsa solution for 30 min. The presence of *Theileria*, *Babesia*, and *Anaplasma* species within erythrocytes was screened using a light microscope. To determine the parasitemia rate, a microscopic field containing *B. aktasi* parasites was randomly selected on the Giemsa-stained peripheral blood smear, and a total of 20 consecutive microscopic fields, including the initially selected field, were examined to determine parasitemia. Microscopic examinations and parasitemia assessment were performed by a single experienced investigator. Blinding was not applied during the microscopic evaluation. The approximate parasitemia rate was then calculated using the formula (number of infected erythrocytes/total number of erythrocytes in the field × 100 = % parasitemia) and expressed as a PPE level, as described previously [17].

A total of 200 µL of EDTA-treated blood samples collected daily for 30 consecutive days from Saanen goats were used for DNA extraction using the PureLink™ Genomic DNA Mini Kit (Invitrogen, Carlsbad, CA, USA), following the manufacturer’s instructions. To evaluate the presence of *B. aktasi* after treatment, all goats were monitored for 30 days using a semi-nested PCR protocol targeting *B. aktasi*-specific primers, as previously described by Ulucesme et al. [36]. Briefly, three *B. aktasi*-specific primers were designed from the 5′ region of the V4 domain of the *B. aktasi 18S rRNA* gene. These included one forward primer, Ba600F (5′-GAATCGACGTTCGTCGTTTA-3′), and two reverse primers, Ba1420R (5′-CCTTTCGGGACAGGATCAAA-3′) and Ba1019R1 (5′-GTTTCAGCCTTGCGACCATACT-3′). For the first round of PCR, each 25 µL reaction mixture contained 2.5 µL of 10× PCR buffer (VitaTaq, Procomcure Biotech GmbH, Bergheim, Austria), 2.5 µL of dNTPs (1.25 mM each), 0.1 µL of Taq DNA polymerase (5 U/µL), 0.5 µL of each outer and inner primer (20 pmol/µL), 2.5 µL of template DNA, and 16.4 µL of nuclease-free water. A 1 µL aliquot of the first-round PCR product was subsequently used as the template for the semi-nested PCR. Amplifications were performed in a Sensequest Labcycler Gradient thermal cycler (Göttingen, Germany). The touchdown PCR protocol and cycling conditions followed a previously described method [36]. In brief, the program began with an initial denaturation at 94 °C for 5 min, followed by cycles of 94 °C for 20 s, 67 °C for 20 s, and 72 °C for 30 s. The annealing temperature was reduced by 2 °C every second cycle until it reached a final “touchdown” temperature of 57 °C.

### 2.9. Statistical Analyses

Changes in hematological parameters (RBC, WBC, HCT, Hb, MCV, RDW-CV, and RDW-SD) in the treated group over time (days 0–15) were statistically analyzed using SPSS version 22.0 (IBM Corp., Armonk, NY, USA). Data normality was assessed using the Shapiro–Wilk test. Depending on the data distribution, one-way repeated-measures ANOVA was used for normally distributed variables, whereas the Friedman test was used for non-normally distributed variables. A *p*-value < 0.05 was considered statistically significant.

## 3. Results

### 3.1. Experimental Infection of the Donor Goats

**Figure 2:**
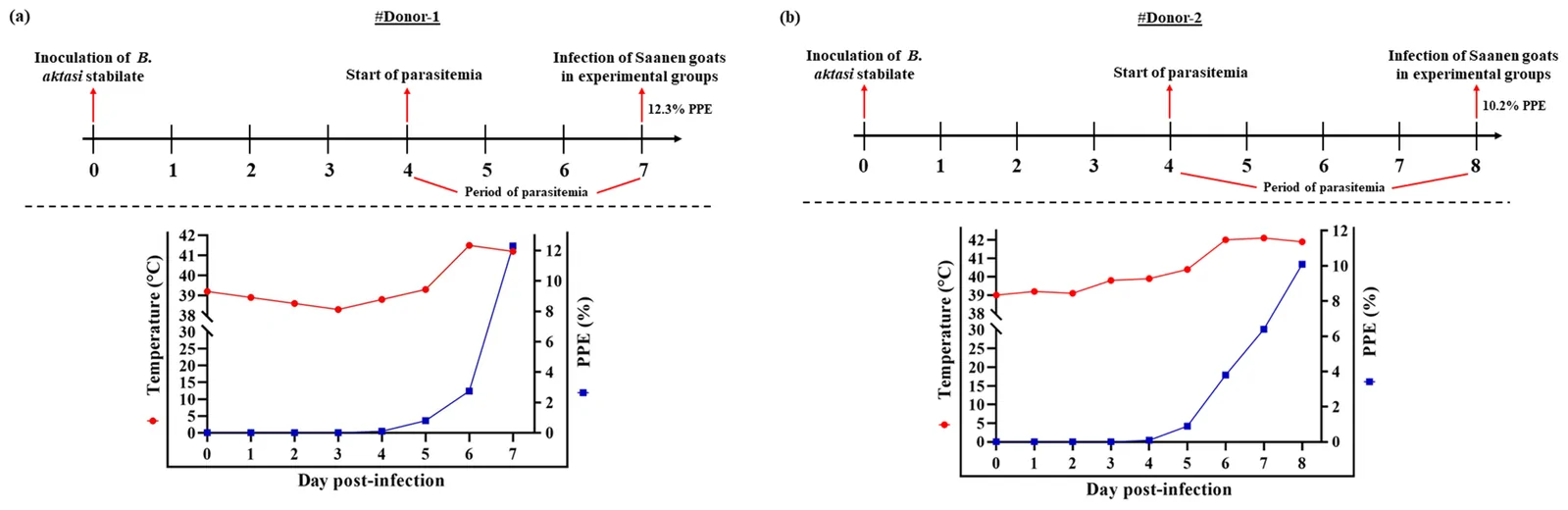
The course of clinical infection in the donor Saanen goats (#Donor-1 and #Donor-2). (**a**) Timeline of clinical infection in the donor goats after inoculation with *B. aktasi* stabilate. (**b**) Course of parasitemia and rectal temperature (°C) changes during infection in the donor goats (on the 7th and 8th days, after the collection of fresh infected blood, the donor Saanen goats were treated and recovered from clinical babesiosis).

Severe infection rapidly developed in the donor goats following intravenous inoculation with the *B. aktasi* stabilate. Main clinical signs of babesiosis such as fever, anemia, and hemoglobinuria were observed. Additional symptoms, including loss of appetite, lethargy, rapid breathing, and immobility, were also observed in the goats. Piroplasm forms of *B. aktasi* were first detected in peripheral blood smears on the 4th day post-inoculation (DPI). In #Donor-1, the rectal temperature peaked at 41.5 °C on the 6th DPI, while parasitemia increased progressively and reached 12.3% on the 7th DPI. In #Donor-2, the rectal temperature peaked at 42.1 °C, and parasitemia reached 10.2% on the 8th DPI. The donor goats recovered from the disease following treatment with IMDP and antibiotics administered on the same day as blood collection for experimental infection of the Saanen goat groups. The duration of clinical infection (rectal temperature and PPE) in the donor goat is presented in Figure 2.

### 3.2. Clinical Course and Treatment of Experimentally Infected Saanen Goats

Parasitological findings obtained in both the treatment and control groups following experimental inoculation with *B. aktasi* and the subsequent development of babesiosis are presented in Table 1.

**Figure 3:**
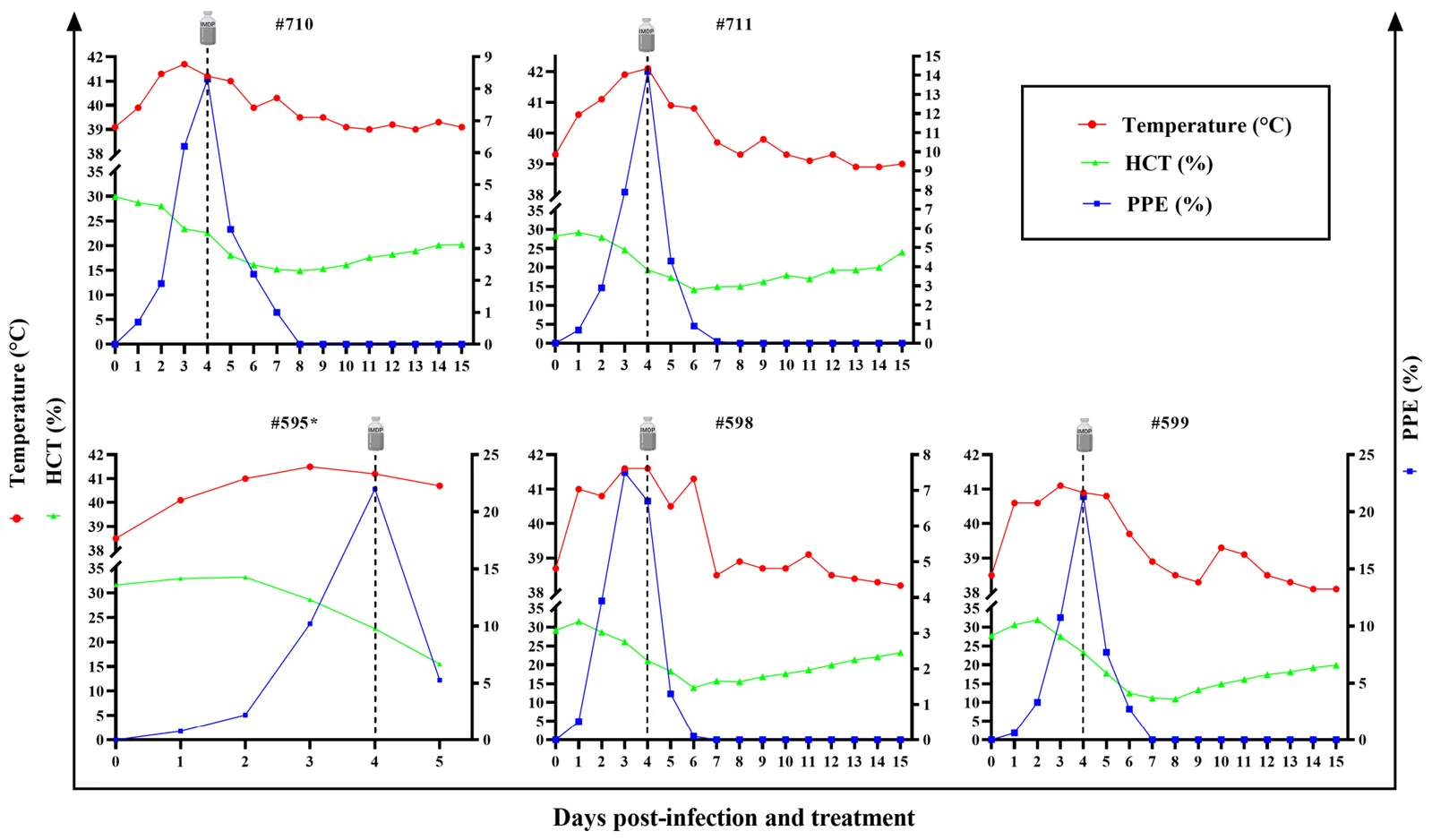
The rectal temperature (°C), HCT (%), PPE (%) dynamics, and response to treatment in experimentally infected Saanen goats. The black dashed lines indicate IMDP treatment. (* died due to severe clinical infection caused by *B. aktasi*).

Following inoculation of the parasite, severe babesiosis developed in the Saanen goats belonging to the treatment group (#595, #598, #599, #710, #711). In all goats, parasites were detected in peripheral blood smears on the 1st DPI. Upon the appearance of intracellular parasites, rectal temperature rapidly increased, reaching a peak of 42.1 °C. The highest PPE levels were recorded as 22%, 7.5%, 21.3%, 8.3%, and 14.2% in goats #595, #598, #599, #710, and #711, respectively. Peak PPE was observed on the 4th DPI in goats #595, #599, #710, and #711, whereas it occurred on the 3rd DPI in goat #598 (Table 1). Hematocrit (HCT) levels began to decrease from the 2nd DPI in goats #595 and #599, and from the 1st DPI in other goats (Figure 3). Typical clinical signs of babesiosis such as fever, anemia, hemoglobinuria and jaundice were observed in the treatment group; however, jaundice was not severe. Hemoglobinuria was detected in all goats starting on the 3rd DPI and worsened on the 4th DPI, along with severe lethargy, complete anorexia, increased immobility, and marked depression with the head lowered and reluctance to move.

Treatment was administered on the 4th DPI, as clinical symptoms such as hemoglobinuria, lethargy, and immobility became more severe. Throughout the post-treatment period, no adverse or toxic effects attributable to IMDP were observed in any of the treated animals. A marked decrease in both temperature and PPE was observed within one day following treatment. However, goat #595 died one day after treatment. In goat #598, a temperature of 41.3 °C was recorded on the 2nd day post-treatment (6th DPI), but returned to normal levels in the following days. No parasites were detected in the peripheral blood smears of goats #598 and #599 from the 3rd day post-treatment, while goats #710 and #711 became microscopically negative from the 4th day post-treatment (Table 1). HCT levels began to increase on the 6th DPI in goat #598 and on the 8th DPI in goats #599–#711, gradually returning to normal ranges (Figure 3).

### 3.3. Experimental B. aktasi Infection Results in Severe Clinical Signs in the Absence of Treatment

After the inoculation with fresh *B. aktasi*-infected blood, severe infection developed in the control group of Saanen goats (#801, #802, #803, #804, #805) resulting in death in four Saanen goats (#801, #802, #803, #805). Parasites were detected in the peripheral blood smears and rectal temperatures began to rise in all goats one day after inoculation. Peak temperatures ranged between 41.2 °C and 42.0 °C (Table 1). All typical clinical signs of babesiosis were observed in the Saanen goats. Severe anemia and hemoglobinuria developed rapidly.

**Figure 4:**
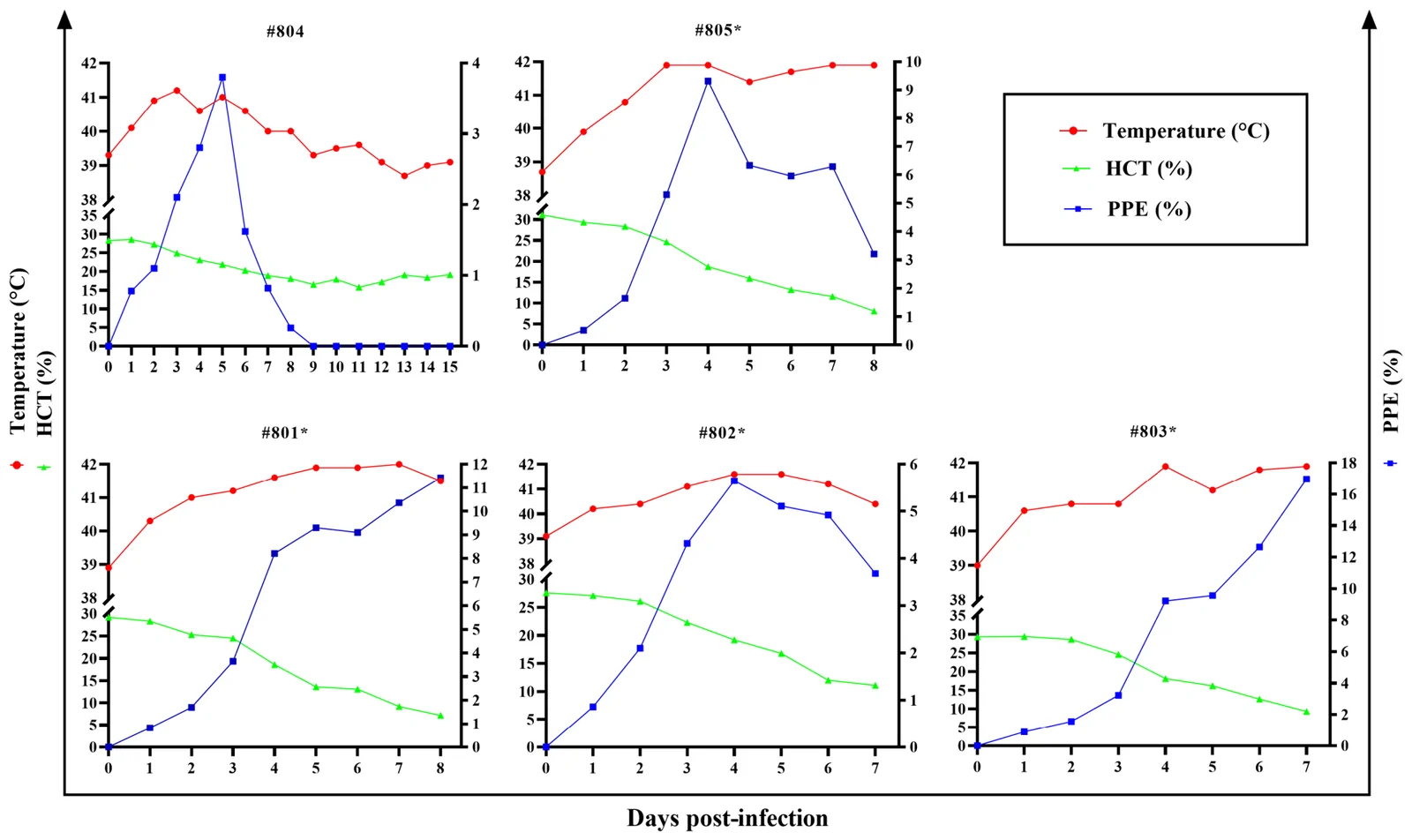
The course of temperature (°C), HCT (%) and PPE (%), and changes in duration of infection in Saanen goats infected with *B. aktasi* in the control group without treatment (* died due to severe clinical infection caused by *B. aktasi*).

In goat #801, a peak PPE of 11.4% was recorded on the 8th DPI. A rectal temperature of 42.0 °C was observed on the 7th DPI, and it died on the 8th DPI. In goats #802 and #805, peak PPE levels of 5.7% and 9.3%, respectively, were recorded on the 4th DPI. Although PPE decreased on the following days, goat #802 died on the 7th DPI, and goat #805 died on the 8th DPI due to severe babesiosis. In goat #803, parasitemia reached a maximum PPE of 17.0% on the 8th DPI. The highest rectal temperature recorded was 41.9 °C on the 7th DPI, and it subsequently died on the 8th DPI. The maximum PPE reached 3.8% in goat #804 and parasites were detected in blood smears for eight consecutive days. After a febrile period lasting 8 days, no parasites were observed in peripheral blood on the 9th DPI, and this goat survived (Table 1). In all control goats, hematocrit (HCT) levels began to decline on the first day post-infection, and in the surviving goat (#804), HCT began to increase again on the 9th DPI (Figure 4).

### 3.4. Alterations in Hematological Parameters Following Experimental B. aktasi Infection in Treated and Control Groups

The statistical analysis of hematological parameters in the treatment group is presented in Table 2. Significant changes were observed in RBC, WBC, HCT, Hb, MCV, RDW-CV, and RDW-SD values over the course of infection and treatment. Overall, significant temporal changes were observed in all hematological parameters during the experimental period. Following IMDP treatment, RBC, HCT, and Hb values showed an increasing trend; however, these increases did not reach statistical significance. In contrast, WBC, MCV, RDW-CV, and RDW-SD changed significantly throughout the study period.

**Figure 5:**
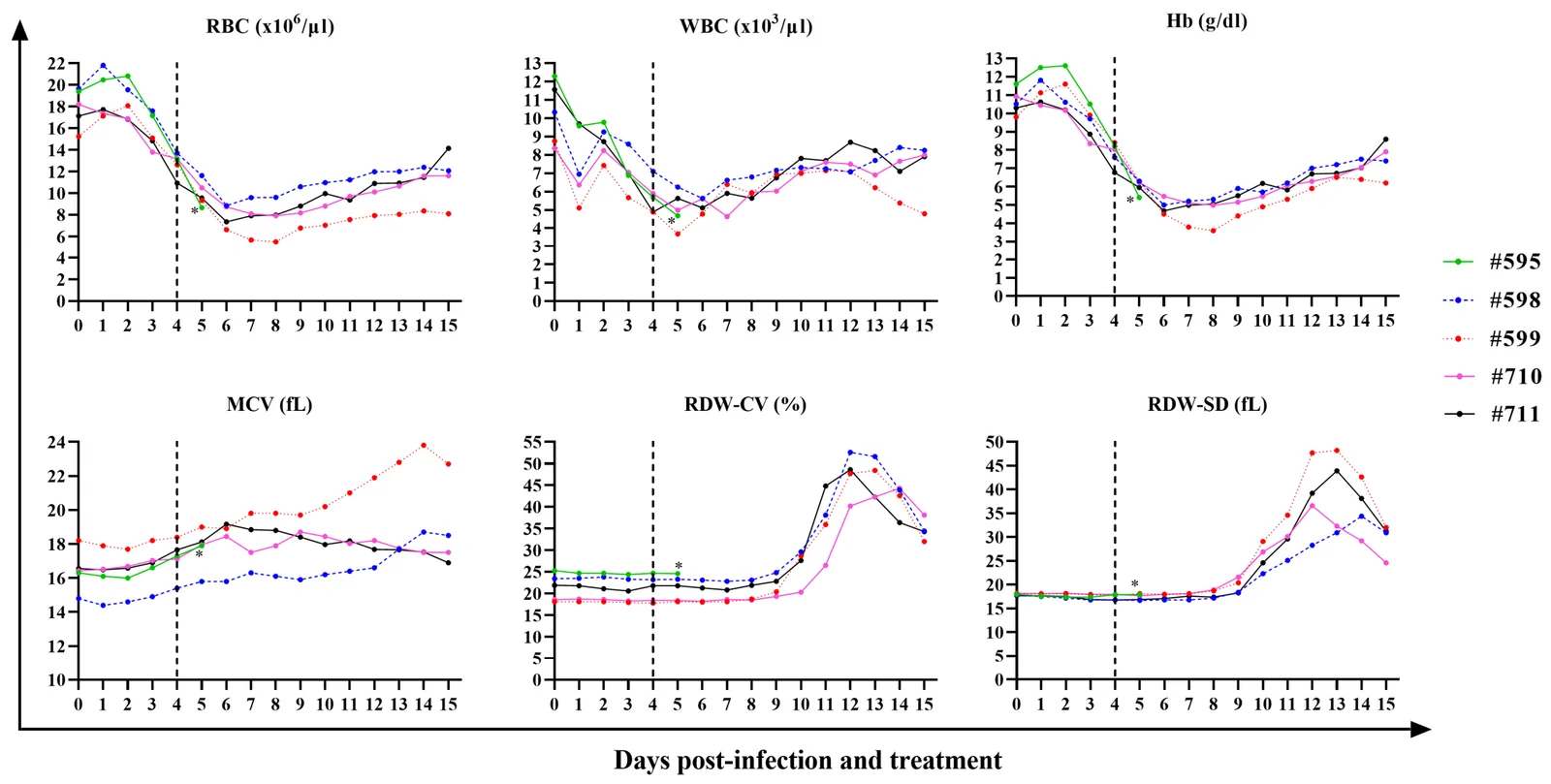
Hematological changes (RBC, Hb, WBC, MCV, RDW-CV, RDW-SD) in *B. aktasi*-infected Saanen goats treated with IMDP. Black dashed lines indicate treatment. Data for goat #595, which died of acute babesiosis, are unavailable after the 5th DPI (* death due to severe infection).

**Figure 6:**
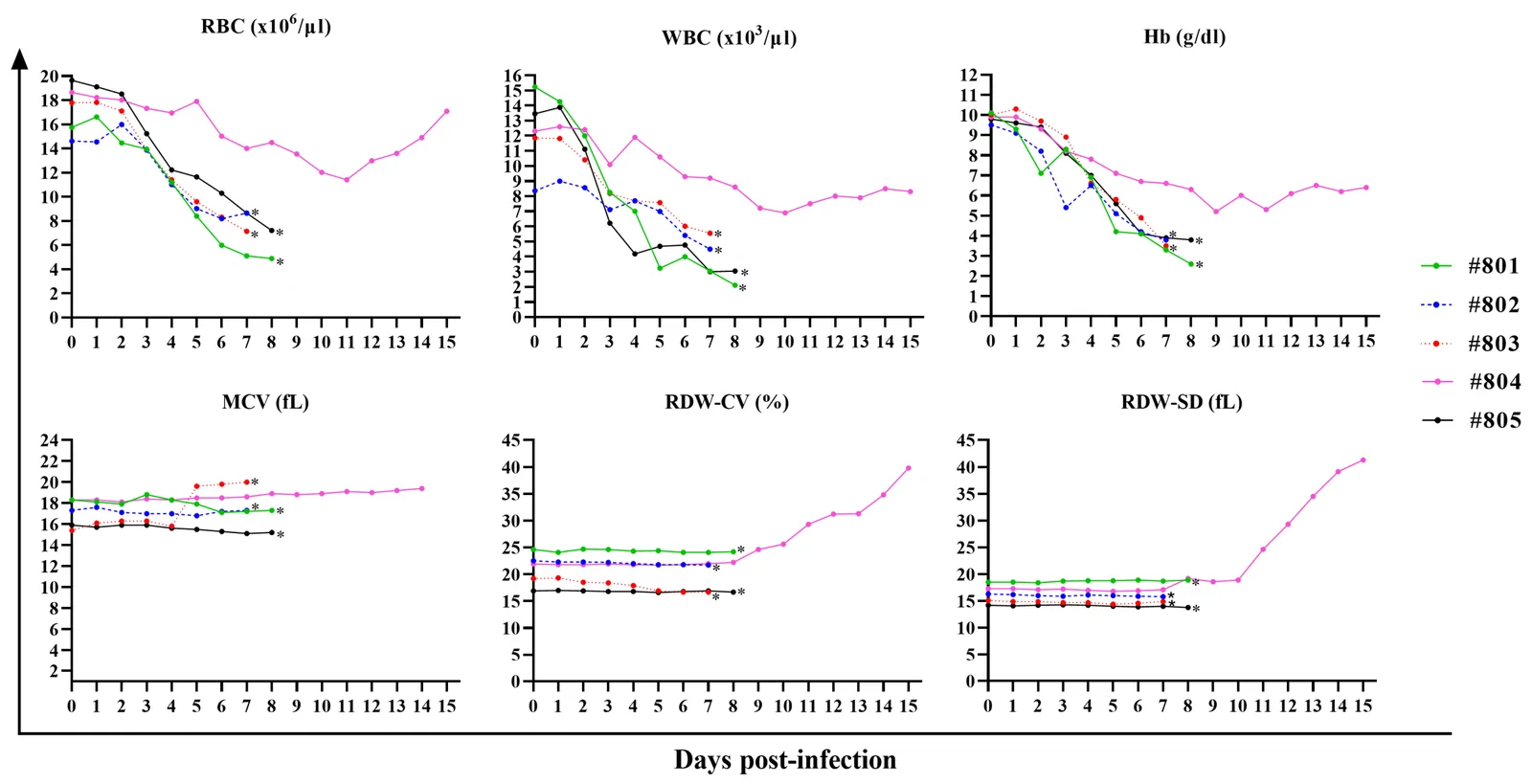
Post-infection trends in RBC, Hb, WBC, MCV, RDW-CV, and RDW-SD of Saanen goats in the control group experimentally infected with *B. aktasi*. Hematological data for goats #801, #802, #803 and #805, which died of acute babesiosis, were unavailable after the 7th and 8th DPI, respectively (* death due to severe infection).

Hematological parameters of experimentally infected Saanen goats with *B. aktasi* are shown for the treatment and control groups in Figure 5 and Figure 6, respectively.

Significant changes were observed in the hematological parameters of Saanen goats in the treatment group following therapy. After inoculation with infected blood, an increase in PPE was accompanied by decreases in RBC and HB levels. However, these decreases stopped 2–4 days after IMDP treatment and began to increase in the following days. WBC levels, which showed a fluctuating decrease until treatment, started to increase 1–2 days post-treatment. No notable changes were observed in RDW-CV and RDW-SD values during the infection period, but a rapid increase was observed five days after treatment. Additionally, except for goats #710 and #711, MCV values showed a gradual increase until the 14th DPI during the course of infection (Figure 5).

In the control group, a rapid decrease in RBC, Hb, and WBC values was observed following the onset of infection compared to pre-infection values. This decline continued until the day of death, occurring on the 7th DPI in goats #802 and #803 and on the 8th DPI in goats #801 and #805. In goat #804, a noticeable decrease was also observed compared to the pre-infection levels in these parameters. From the 9th DPI, when no parasites were observed in peripheral blood smears, these parameters gradually increased and returned to normal ranges in goat #804. In the control group of Saanen goats, no overall changes were observed in MCV, RDW-CV, and RDW-SD values compared with pre-infection levels in any goat except #803. In goat #803, MCV values began to increase on the 4th DPI, followed by RDW-CV values on the 8th DPI and RDW-SD values on the 10th DPI (Figure 6).

### 3.5. Persistence of Babesia aktasi DNA Following IMDP Treatment in Saanen Goats

*Babesia aktasi* DNA remained detectable by semi-nested PCR in blood samples collected daily from the four surviving Saanen goats (#598, #599, #710 and #711) in the treatment group for up to 30 days post-treatment. A consistent PCR positivity was observed throughout the entire one-month period.

## 4. Discussion

In this study, which represents the first evaluation of treatment for *B. aktasi*, a parasite known to be highly pathogenic in Saanen goats, administration of IMDP (1.2 mg/kg) resulted in marked clinical improvement, microscopic clearance of parasitemia, and recovery of most treated animals. However, *B. aktasi* DNA remained detectable by semi-nested PCR throughout the 30-day follow-up period, indicating that although IMDP effectively controlled the clinical disease, complete parasitological clearance could not be demonstrated.

Experimental infections established via inoculation of fresh *B. aktasi*-infected blood resulted in severe babesiosis in both the treatment and control groups. In the control group, peak PPE levels ranged between 3.8% and 17.0%, whereas in the treatment group, PPE reached levels between 7.5% and 22%. In contrast, following IMDP treatment, four of the five goats in the treatment group exhibited a rapid reduction in parasitemia, followed by microscopic clearance, complete resolution of clinical signs, and recovery from the disease. A previous study conducted in Saanen goats similarly reported PPE levels ranging from 4.3% to 33.5% in the immunosuppressed group and from 2.1% to 7.6% in the immunocompetent group, with the infections resulting in fatal outcomes [18]. These findings support the conclusion that *B. aktasi* infection exhibits high virulence in purebred Saanen goats.

Experimental infection with *B. aktasi* in immunocompetent Saanen goats results in a highly severe disease that may lead to death and presents with characteristic clinical signs of babesiosis [18]. Similarly, all goats in both the control group and the treatment group (prior to therapy) exhibited the full spectrum of clinical signs associated with acute babesiosis. These included high fever, anemia, hemoglobinuria, mild jaundice, anorexia, lethargy, and weakness. One goat in the control group recovered spontaneously without any treatment. This finding shows similarity to previous studies. Ulucesme et al. [18] reported that Saanen goats in the immunocompetent group infected with *B. aktasi* exhibited severe clinical signs but recovered without the need for treatment. Similarly, in another study, three healthy sheep experimentally infected with *B. ovis* also recovered from the infection without treatment, despite parasitemia reaching 6.3% and the presence of marked clinical signs of babesiosis [9]. In the treatment group, clinical signs began to subside as early as one day after administration of IMDP. Notably, hemoglobinuria resolved rapidly, and four out of five goats responded successfully to treatment.

The efficacy and recommended dosage of IMDP for the treatment of *B. ovis* infections in sheep have been previously reported [24,26,37]. Sevinc et al. [26] demonstrated that in splenectomized sheep experimentally infected with *B. ovis*, administration of IMDP at a therapeutic dose (1.2 mg/kg) resulted in the complete disappearance of visible parasites on blood smears 48 h after treatment in five out of six animals. In the remaining case, parasitemia recurred, but the infection was successfully cleared following a second dose. In our study, no parasites were detected in blood smears from goats #598 and #599 from the 3rd day after treatment, whereas no parasites were detected in goats #710 and #711 from the 4th day after treatment. This result indicates that IMDP elicits a rapid therapeutic response in infections caused by *B. aktasi*, similar to its effect in *B. ovis* infections. In a subsequent field study, Sevinc et al. [8] reported that while IMDP was generally effective in treating naturally infected *B. ovis* cases, post-treatment mortality was observed in some animals. In this study, the dose used for treatment was 1.2 mg/kg, which is the standard dose applied in sheep. In our study, although IMDP treatment was generally effective, one of the five treated Saanen goats died as a result of acute babesiosis caused by *B. aktasi*. This finding raises the possibility that alternative dosing regimens may be needed to reduce the risk of fatal outcomes. These results suggest that although IMDP exhibits high therapeutic efficacy, factors such as disease severity, timing of administration, and the physiological status of the host may influence treatment outcomes. The results in the control group were consistent with those reported by Sevinc et al. [26], as all goats developed severe babesiosis and four of the five animals died.

Hematological parameters such as HCT, RBC, and HGB decreased in both groups as PPE increased, consistent with a hemolytic anemia resulting from infection-related erythrocyte destruction [8,38,39]. Similar findings were reported in previous pathogenicity studies conducted in Saanen goats, where these values decreased proportionally with rising PPE [18]. In the surviving goats of the treatment group, these parameters gradually returned to normal ranges in the following days, a pattern consistent with a regenerative erythropoietic response following resolution of hemolysis. This regenerative response was further supported by the concurrent increase in RDW values observed after treatment, which reflects the release of immature, variably sized erythrocytes (reticulocytes) into circulation as the bone marrow compensates for anemia [40]. Changes in MCV values were less consistent and should therefore be interpreted cautiously, particularly given the limited observation period and small sample size. In sheep infected with *B. ovis* and exhibiting moderate anemia, a similar recovery of hematological values following IMDP treatment has also been reported [8]. In the single surviving goat of the control group, these values likewise returned to normal levels following spontaneous recovery. In both groups, WBC levels dropped rapidly after infection, possibly reflecting the systemic inflammatory and stress responses associated with acute babesiosis; the subsequent increase in WBC count observed in the treatment group after therapy is consistent with resolution of infection and a return toward normal myeloid activity, a pattern also reported in *B. ovis*-infected sheep after treatment [8]. Overall, the recovery kinetics of these hematological parameters in surviving animals suggest that, once parasitemia is cleared microscopically, bone marrow regenerative capacity is sufficient in restoring hematological homeostasis within the study period, even though molecular evidence of parasite persistence remained.

Following treatment, semi-nested PCR positivity persisted for 30 days in four goats (#598, #599, #710 and #711) in the treatment group, indicating persistent detection of parasite DNA after treatment. Similarly, previous studies have demonstrated the limited efficacy of IMDP in completely eliminating certain piroplasm infections [27,28]. Despite repeated treatment regimens, IMDP has been shown to be ineffective in fully eliminating *T. equi* infections in horses, and treatment has also failed in horses infected with *T. haneyi* [27]. Similarly, IMDP treatment was unsuccessful in dogs infected with *B. negevi*, with the parasite reported to persist in the host for up to seven months [28].

The present study has several limitations that should be considered when interpreting the findings. First, although the experimental design allowed evaluation of the therapeutic response to IMDP, the relatively small sample size may have limited the statistical power and generalizability of the results. Therefore, future studies including larger treatment and control groups are warranted to further validate the therapeutic efficacy of IMDP against *B. aktasi* infection. Second, although persistent detection of *B. aktasi* DNA was monitored using semi-nested PCR, future studies incorporating quantitative PCR (qPCR) would enable quantification of parasite burden over time and provide a more comprehensive assessment of treatment response and parasite persistence. Finally, molecular follow-up was limited to 30 days after treatment. Consequently, it remains unclear whether the persistent PCR positivity reflected viable parasites or residual parasite DNA, whether PCR positivity would eventually resolve, or whether recrudescence could occur beyond the observation period. Future studies including larger sample sizes, quantitative molecular techniques, and longer-term follow-up are warranted to clarify these issues and to optimize therapeutic strategies for *B. aktasi* infection.

## 5. Conclusions

IMDP treatment (1.2 mg/kg) was generally effective in achieving clinical improvement, microscopic clearance of parasitemia, and recovery of hematological parameters in most treated animals. However, the persistence of *B. aktasi* DNA throughout the 30-day follow-up period indicates that complete parasitological clearance could not be demonstrated. Therefore, while IMDP appears to be an effective option for the clinical management of acute *B. aktasi* infection, longer-term follow-up studies incorporating quantitative molecular techniques are needed to determine whether persistent PCR positivity reflects viable parasites, to evaluate the potential for recrudescence, and to optimize future treatment protocols.

## Figures and Tables

**Table 1:** Parasitological and clinical findings in imidocarb dipropionate-treated (treatment group) and untreated (control group) Saanen goats experimentally infected with *Babesia aktasi*.

Treatment Group (*n* = 5)	Prepatent Period (Days)	Max. Fever (°C)	Max. PPE (%)	PPE Duration (Days)	Day of Death (Days)
#595	1	41.5	22.0	5	5
#598	1	41.6	7.5	6	survived
#599	1	41.1	21.3	6	survived
#710	1	41.7	8.3	7	survived
#711	1	42.1	14.2	7	survived
Control Group (*n* = 5)					
#801	1	42.0	11.4	8	8
#802	1	41.6	5.7	7	7
#803	1	41.9	17.0	7	7
#804	1	41.2	3.8	8	survived
#805	1	41.9	9.3	8	8

**Table 2:** Statistical analysis of hematological parameters in IMDP-treated Saanen goats experimentally infected with *Babesia aktasi* at baseline (Day 0), treatment (Day 4), and post-treatment (Day 15).

Hematological Parameters	Initial (Day 0) Mean ± SD Median (Min–Max)	4th Day Mean ± SD Median (Min–Max)	15th Day Mean ± SD Median (Min–Max)	*p*-Values
RBC (×10^6^/µL)	17.92 ± 1.80 ^a^ 18.20 (15.25–19.65)	12.71 ± 1.06 ^b^ 13.12 (10.94–13.70)	11.79 ± 2.28 ^b^ 12.08 (8.11–14.15)	*p* = 0.0035
WBC (×10^3^/µL)	10.26 ± 1.71 ^a^ 10.33 (8.36–12.29)	5.69 ± 0.90 ^b^ 5.68 (4.89–7.10)	7.39 ± 1.46 ^ab^ 7.99 (4.79–8.25)	*p* = 0.0085
HCT (%)	29.32 ± 1.52 ^a^ 29.10 (27.70–31.60)	21.78 ± 1.59 ^b^ 22.60 (19.30–23.20)	21.82 ± 1.80 ^b^ 21.82 (19.90–24.00)	*p* = 0.0002
HB (g/dL)	10.62 ± 0.67 ^a^ 10.50 (9.80–11.60)	7.80 ± 0.64 ^b^ 8.03 (6.77–8.40)	7.64 ± 0.91 ^b^ 7.90 (6.20–8.59)	*p* = 0.0003
MCV (fL)	16.46 ± 1.20 ^b^ 16.43 (14.80–18.20)	17.18 ± 1.10 ^ab^ 17.30 (15.40–18.40)	18.97 ± 2.27 ^a^ 18.50 (16.90–22.70)	*p* = 0.0185
RDW-CV (%)	21.46 ± 3.08 ^b^ 21.90 (18.10–25.30)	21.18 ± 3.00 ^b^ 21.80 (17.80–24.70)	34.78 ± 2.19 ^a^ 34.40 (32.00–38.10)	*p* = 0.0013
RDW-SD (%)	17.96 ± 0.20 ^ab^ 18.00 (17.70–18.20)	17.46 ± 0.60 ^b^ 17.80 (16.80–18.00)	29.67 ± 2.95 ^a^ 30.90 (24.60–32.00)	*p* = 0.0008

Initial: Day 0, Treatment: 4th day, post-treatment: 15th day. The normality of the data was assessed using the Shapiro–Wilk test. Depending on the data distribution, changes over time were analyzed using one-way repeated-measures ANOVA for normally distributed variables (RBC, Hb, MCV, RDW-CV, and HCT) and the Friedman test for non-normally distributed variables (WBC and RDW-SD). Different superscript letters (a and b) within the same row indicate statistically significant differences between time points (*p* < 0.05).

## Data Availability

The original contributions presented in the study are included in the article, further inquiries can be directed at the corresponding author.
